# Animal models of functional HIV cure

**DOI:** 10.1186/1742-4690-9-S1-I9

**Published:** 2012-05-25

**Authors:** Guido Silvestri

**Affiliations:** 1 Emory University School of Medicine, Atlanta, USA

## 

Developing new therapeutic strategies to achieve a functional cure for HIV infection is a central objective for contemporary AIDS research. A key obstacle towards a functional cure for HIV/AIDS is the paucity of information regarding the establishment and maintenance of the persistent reservoirs of latently HIV-infected cells which support the rapid reactivation of virus replication when antiretroviral therapy (ART) is stopped. Given the many and important similarities between HIV-infection of humans and SIV infection of macaques, it is very likely that crucial information on the nature of HIV reservoirs may come from studies conducted in SIV or SHIV-infected macaques that are treated with ART. While this area of AIDS research is still in its early stages, it is important to note that these studies have now become possible due to the availability of antiretroviral regimens that successfully reduce SIV or SHIV replication below detectable limits. Specific advantages of the non-human primate models for HIV latency, reservoirs and functional cure include the following: (i) possibility to conduct extensive characterization of the virus reservoirs and pathogenic processes in tissues (including elective necropsy); (ii) performance of in vivo pilot trials of new therapeutic approaches can be conducted in a timely and controlled fashion; (iii) developing and testing of “risky” interventions (i.e., cell depletion experiments, stem cell-based interventions etc) that may pose significant ethical challenges in humans; and (iv) possibility to control for various clinical parameters that are very hard to control in humans (time of infection, duration of ART etc). On the other hand, current limitations to this type of studies are their cost and the lack of standardized research resources (i.e., assays, virus stocks, etc). In this presentation, the potential and limitations of animal models of functional HIV cure will be discussed together with selected examples of therapeutic approaches that could be developed and tested in non-human primates.

